# The Sleep Or Mood Novel Adjunctive therapy (SOMNA) trial: a study protocol for a randomised controlled trial evaluating an internet-delivered cognitive behavioural therapy program for insomnia on outcomes of standard treatment for depression in men

**DOI:** 10.1186/s12888-015-0397-x

**Published:** 2015-02-05

**Authors:** Nicole L Cockayne, Helen M Christensen, Kathleen M Griffiths, Sharon L Naismith, Ian B Hickie, Frances P Thorndike, Lee M Ritterband, Nick S Glozier

**Affiliations:** Healthy Brain Ageing Program, Brain and Mind Research Institute, University of Sydney, 94 Mallett Street, Camperdown, NSW 2050 Australia; Black Dog Institute, University of New South Wales, Prince of Wales Hospital, Hospital Road, Randwick, NSW 2031 Australia; National Institute for Mental Health Research, The Australian National University, Building 63, Canberra, ACT 0200 Australia; Behavioral Health and Technology Laboratory, Department of Psychiatry and Neurobehavioral Sciences, The University of Virginia, PO Box 800623, Charlottesville, VA 22908 USA

**Keywords:** Depression, Insomnia, Adjunctive, e-health, Cognitive behavioural therapy, Randomised controlled trial, Men

## Abstract

**Background:**

Insomnia is a significant risk factor for depression onset, can result in more disabling depressive illness, and is a common residual symptom following treatment cessation that can increase the risk of relapse. Internet-based cognitive behavioural therapy for insomnia has demonstrated efficacy and acceptability to men who are less likely than women to seek help in standard care. We aim to evaluate whether internet delivered cognitive behavioural therapy for insomnia as an adjunct to a standard depression therapeutic plan can lead to improved mood outcomes.

**Methods/Design:**

Male participants aged 50 years or more, meeting Diagnostic and Statistical Manual of Mental Disorders criteria for current Major Depressive Episode and/or Dysthymia and self-reported insomnia symptoms, will be screened to participate in a single-centre double-blind randomised controlled trial with two parallel groups involving adjunctive internet-delivered cognitive behavioural therapy for insomnia and an internet-based control program. The trial will consist of a nine-week insomnia intervention period with a six-month follow-up period. During the insomnia intervention period participants will have their depression management coordinated by a psychiatrist using standard guideline-based depression treatments. The study will be conducted in urban New South Wales, Australia, where 80 participants from primary and secondary care and direct from the local community will be recruited. The primary outcome is change in the severity of depressive symptoms from baseline to week 12.

**Discussion:**

This study will provide evidence on whether a widely accessible, evidence-based, internet-delivered cognitive behavioural therapy for insomnia intervention can lead to greater improvements than standard treatment for depression alone, in a group who traditionally do not readily access psychotherapy. The study is designed to establish effect size, feasibility and processes associated with implementing e-health solutions alongside standard clinical care, to warrant undertaking a larger more definitive clinical trial.

**Trial registration:**

Australian and New Zealand Clinical Trials Registry ACTRN12612000985886.

## Background

Symptoms of insomnia are commonly comorbid with depression, particularly in older adults [1]. In this population, although advanced sleep phase can occur, insomnia is the most commonly reported sleep disturbance; this includes complaints of difficulty falling asleep, frequent nocturnal awakenings, and early morning wakefulness [2,3]. These lead to shorter overall sleep duration, which in turn contributes to chronic patterns of mental ill health [4]. Epidemiological studies suggest that insomnia is a significant risk factor for depression onset and recurrence in both younger and older samples [5-7]. Insomnia comorbid with depression is indicative of a more severe illness and, despite its implications, tends to be under-treated [5]. In late life depression, sleep disturbance appears to be most prominent at the onset of the episode [8] and has been shown to be a prognostic marker [9,10]. Insomnia may be separate or reflect an inadequately treated episode of Major Depressive Disorder (MDD). Reports of the prevalence of residual insomnia symptoms in those responding to treatment for MDD, range from 30% up to 90% [11]. Importantly, failure to achieve full remission from even the first episode of MDD is a strong predictor of recurrence and chronicity as shown in landmark National Institute of Mental Health studies [12] and confirmed in the STAR-D trial [13]. Further, insomnia is the most common residual symptom after completion of drug or therapeutic treatment of mood disorder [11,12] occurring in up to two thirds of this group in the STAR-D study [13]. Thus insomnia in elderly people with previous MDD is a strong and independent predictor of recurrent [10] and persisting MDD [9].

The question of whether adjunctive treatment of insomnia can improve the standard treatment of depression was addressed in a pilot trial by Manber et al. [14]. They reported that the addition of seven sessions of cognitive behavioural therapy for insomnia (CBTi) to escitalopram resulted in a higher rate of remission of depression (61.5%) than in the control arm (33.3%). The adjunctive therapy was also associated with a greater remission from insomnia (50.0%) as compared to control (7.7%) and larger improvement in all diary and actigraphy measures of sleep, except for total sleep time. More recently, Watanabe et al. [15] demonstrated that four weekly face-to-face sessions of CBTi as a supplement to outpatient treatment in psychiatric clinics was associated with higher rates of remission of depression (50.0%) compared with treatment as usual (6.0%), with similarly favourable rates of remission for insomnia (50.0% versus 0.0%). This study has since been extended by Wagley et al. [16] using a briefer version of face-to-face CBTi in a broader sample of patients with psychiatric disorders, which has also shown significant reductions in both depressive symptoms and sleep quality following treatment.

Men with symptoms of depression and anxiety, particularly in the oldest and youngest age groups seek help far less frequently than women for a given threshold or condition [17]. Whilst help-seeking for depression remains low, over half of people with insomnia seek treatment in primary care [18] making it likely that an insomnia intervention program would be well received and utilised. Internet-based programs have some distinct advantages in the delivery of treatment and prevention services. Treatment using e-health applications is 20 times more cost effective than face-to-face psychological treatment and 50 times more efficient than medication in terms of disability-adjusted life years averted [19]. However, we are not aware of any study evaluating the use of adjunctive internet-based CBTi in older men with depression.

With the development and initial evaluation of Sleep Healthy Using the Internet (SHUTi), members of our team have demonstrated the efficacy of an internet intervention for adults with insomnia [20]. This intervention is firmly grounded in the research supporting the efficacy of face-to-face CBTi [21], including the primary components of sleep restriction, stimulus control, cognitive restructuring, sleep hygiene, and relapse prevention. SHUTi is an interactive, self-help program, which is fully automated and based on principles of self-help. In this community-based study of adults with insomnia [20], participants randomly assigned to receive SHUTi not only experienced significant sleep improvements, but also demonstrated improvements in comorbid psychological symptoms and quality of life, compared to those in a wait-list control group [22].

As such, we hypothesise that providing SHUTi as an adjunct to standard care in older men with comorbid depression and insomnia symptoms will improve depression more than an adjunctive controlled website providing insomnia education alone.

### Objectives

In older men with major depression, this trial aims to:

1. Investigate the efficacy of SHUTi as an adjunctive treatment to standard *beyondblue* guideline-based treatment [23,24] for reducing depression symptom severity;
2. Determine whether adjunctive SHUTi decreases anxiety and insomnia symptom severity;
3. Ascertain whether SHUTi is associated with changes in actigraphy/sleep diary determined sleep in people with comorbid depression and insomnia symptoms;
4. Explore the processes, predictors and mediators of treatment response and of adherence to SHUTi by determining user characteristics and behaviours that may predict positive outcomes, adherence, trial drop-out, and program acceptability.

## Methods/Design

### Trial design

**Figure 1:**
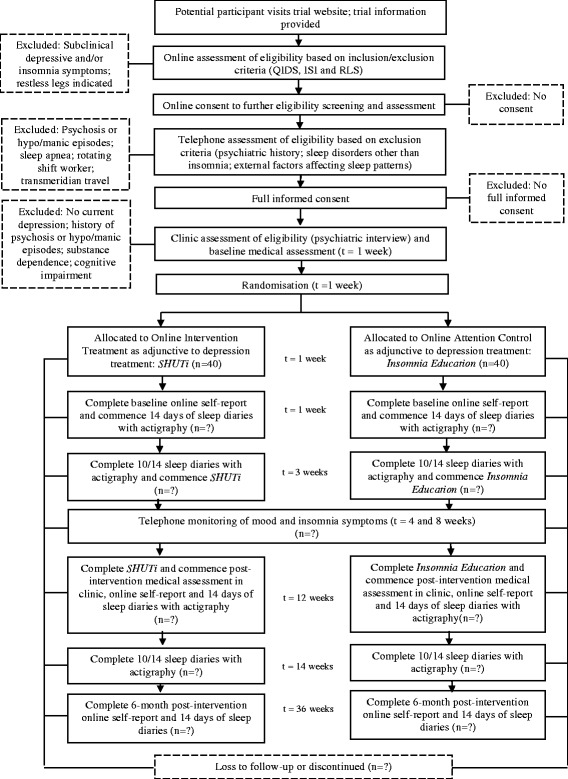
**Proposed flow of participants.**

The study is a single-centre double-blind randomised controlled trial (RCT) with two parallel groups involving an internet-delivered CBTi intervention and an insomnia education control condition. The trial consists of a nine-week insomnia intervention period and six-month follow-up period. The intervention will be delivered as an adjunct to standard guideline-based treatment for depression. As such, during the intervention period, participants in both arms will have their depression assessed, and the treatment managed, by a psychiatrist in accordance with clinical practice guidelines [23,24]. At trial entry, and after randomisation, participants will complete baseline clinical, sleep and self-report measures over two-weeks. They will then commence their internet-delivered CBTi program / web-based insomnia education (control) and continue with it over the subsequent nine-weeks. Participants will be contacted by telephone at Week 4 and Week 8 for trial monitoring purposes. At Week 12, they will commence the post-intervention assessment and will repeat the clinical, sleep and self-report measures collected at baseline. Throughout this period they will have face-to-face sessions with the psychiatrist, their General Practitioner (GP) and other health professionals, as clinically required, to manage their depression. Six months after completion of the post-intervention assessment, participants will complete the self-report, psychological and sleep measures. As such there will be three principal occasions of measurement over the full study period: baseline, post-intervention, and 6-months post-intervention, and the primary endpoint will be a reduction in depressive symptoms at post-intervention (week 12). The total trial period will be nine months. The trial has been registered on the Australian and New Zealand Clinical Trial Registry (ACTRN12612000985886). The trial design is shown graphically in Figure 1.

### Study setting

The target population for the SOMNA trial is Australian male adults, aged 50 years and over, who meet diagnostic criteria for a current depressive disorder and self-reported sleep disturbance. Participants will be recruited from local primary and secondary care providers, as well as directly from the local community via newspaper and radio advertisements, community organisation newsletters and events. An online recruitment and marketing campaign will also be implemented through help-seeking websites and social media.

### Eligibility criteria

Participants eligible for the SOMNA trial must comply with all of the following at the point of randomisation:

1. Have a depressive disorder, defined as screening positive (score greater than or equal to 8) on the Quick Inventory of Depression Symptomology Self Report (QIDS-SR) [25] at preliminary screening, and meeting Diagnostic and Statistical Manual of Mental Disorders (DSM-IV) criteria for current Major Depressive Episode and/or Dysthymia at clinical interview using the Structured Clinical Interview for DSM-IV (SCID) [26];
2. Report insomnia symptoms above threshold (score greater than or equal to 8) on the Insomnia Severity Index (ISI) [27] at preliminary screening; and,
3. Have provided written informed consent before any trial procedures are undertaken.

Participants will be excluded from participating in the SOMNA trial if at the point of randomisation they:

1. Have a prior history of psychosis or hypo/manic episodes, determined either during phone screening or at clinical interview;
2. Have current substance dependence, determined at clinical interview;
3. Score less than 24 on the Mini Mental State Examination (MMSE) [28], determined at clinical interview;
4. Are a rotating shift worker with overnight shifts;
5. Have undertaken transmeridian travel in the past month, where the time difference is greater than two hours and there has been an inadequate period to readjust to local time (adjustment defined as requiring 48 hours for every one hour time difference);
6. Meet criteria for Restless Legs Syndrome (RLS) as defined using the Cambridge-Hopkins RLS short form diagnostic questionnaire [29];
7. Are at high risk for obstructive sleep apnea (OSA) as defined by the Berlin Questionnaire [30], or have been treated for sleep apnea.

### Interventions

All eligible participants will be randomised on a 1:1 basis to receive either SHUTi or the web-based insomnia education control program.

#### Active treatment arm: SHUTi

The Sleep Healthy Using the Internet (SHUTi, http://shuti.me) program is firmly grounded in research supporting the efficacy of face-to-face Cognitive Behavioural Therapy (CBT) for insomnia [20]. Described in detail elsewhere [31], SHUTi consists of six core modules addressing behavioural, educational and cognitive techniques, incorporating sleep restriction and stimulus control, sleep hygiene, cognitive restructuring and relapse prevention. Content is presented in an interactive format featuring text, graphics, audio and animations. The program utilises vignettes and quizzes to deliver information, and provides individually tailored recommendations to help improve sleep as informed from sleep diary information submitted by users. The six core modules are available to complete sequentially and completion involves up to an hour per week, over a nine-week period. Participants are also encouraged to submit daily sleep diaries each week. An automated email notifies participants that a new module is available for completion. At the beginning of each new module, participants receive recommendations for sleep restriction treatment based on their completion of the previous weeks online daily sleep diaries, along with a review of relevant information from previously completed modules. Participants are able to move forward to the next module one week after the previous module is completed in full. Automated midweek reminders to enter sleep diaries, implement strategies and commence the next module are also sent. Each module remains available once it has been completed so participants may review all program content.

#### Control arm

The insomnia education control program provides credible sleep health information addressing the impact and prevalence of insomnia, along with common causes and symptoms of insomnia. It provides limited sleep hygiene information pertaining to lifestyle and environmental factors that may affect sleep. While behavioural and cognitive techniques are mentioned in the control program, unlike SHUTi, the strategies are not obtainable in a tailored, metered, or interactive fashion, but rather the content is presented in a basic text format only. Consideration of the content requires approximately 30 minutes. However, the insomnia education program (but not sleep diaries) is available for further review by participants at any time over the nine-week intervention period.

### Clinical treatment for depression

As part of standard treatment for depression, all participants will undergo a clinical assessment with a psychiatrist prior to study commencement, whereupon a depression treatment plan will be prepared in accordance with clinical practice guidelines [23,24]. This will include watchful waiting, referral to psychotherapy or other interventions such as couple therapy, or medication use under standard collaborative care with the patient’s GP. The assessment will include the Mood Episode and Disorder modules, and the Substance Abuse screens of the SCID, standard clinician rated measures: the 17-item Hamilton Rating Scale for Depression (HAM-D) [32], and the Hamilton Rating Scale for Anxiety (HAM-A) [33], Mini Mental State Examination [28], Global Assessment of Functioning (GAF) [34], Social and Occupational Functioning Assessment Scale (SOFAS) [35] and Cumulative Illness Rating Scale for Geriatrics (CIRS-G) [36] as well as completion of a full psychiatric and medical history. After two weeks, participants will be required to undergo a clinical review with the psychiatrist, in accordance with their treatment plan. Over the subsequent nine weeks, participants will undergo further psychiatric review and management as clinically required. Standard clinical instruments will be administered to monitor depressive symptoms over this period. If any changes to medication dosage are required, this will be discussed between the patient, trial psychiatrist and/or referring doctor. At the end of the trial period the participant will be referred back to their GP or treating doctor, or if required, managed in the trial clinic until appropriate for discharge.

### Participant monitoring and program adherence

While individual completion of SHUTi and the insomnia education control programs will be systematically tracked using methods embedded in the website infrastructure, their completion (or otherwise) will be achieved without any direct supervision or encouragement from the trial team. Participants will be able to seek technical support as needed (e.g. if a username or password is forgotten) but as this is a trial of an adjunctive insomnia treatment, no specific clinical support will be provided. Furthermore, as all participants will be under the clinical care of their treating psychiatrist, safety and general monitoring will be conducted at each scheduled clinical review and follow-up appointment(s). Research assistants will contact all participants by telephone at Week 4 and Week 8 (as indicated in Figure 1) whereby data will be collected against the primary and secondary outcome measures, addressing mood state and insomnia symptoms. Participants demonstrating deterioration in mood state will be referred to their treating psychiatrist for risk management and follow-up.

### Self report measures

#### Primary outcome

The primary outcome is change in severity of self-reported depressive symptoms from baseline to three months, as measured by the Centre for Epidemiological Studies Depression scale (CES-D) [37]. The 20 items in the CES-D measure symptoms of depression over the past week on a 0–3 scale, and provide a summary score ranging from 0 to 60 with higher scores indicating the presence of greater depressive severity. Although originally created for the general population, the CES-D is a reliable and valid measure of depressive symptoms and widely used in clinical populations [38]. A self-report measure, the CES-D will be embedded into each of the formal assessments at baseline, post-intervention and six-month post-intervention follow-up. The change in severity of clinician-rated depressive symptoms over a three-month period, will also be determined using the HAM-D [32]. Considered the gold standard depression assessment tool, the HAM-D is the most widely used clinician-administered scale in clinical trials of antidepressant treatment. It rates the severity of symptoms observed in depression such as low mood, insomnia, agitation, anxiety and weight loss, with higher scores indicative of more pronounced symptomatology. Mean changes in continuous depressive symptom scores from baseline will be compared between the two randomised groups at three-months. Change scores will be based on summed scores derived from the CES-D and the HAM-D respectively.

#### Secondary outcomes

Secondary outcomes will comprise changes in the severity of self-reported anxiety and insomnia symptoms respectively. Anxiety symptoms will be assessed measured by the State Trait Personality Inventory (STPI – Trait only) [39]. The STPI is a self-administered questionnaire designed to measure dispositional anxiety, anger, depression and curiosity in adults i.e. how they “generally feel”. It consists of four 10-item subscales with each item rated on a four-point frequency scale. Higher scores are positively correlated with higher levels of dispositional emotional states. The STPI is a widely used measure and has high validity and reliability, particularly for the anxiety and anger scales [39]. Mean changes in total summed scores from baseline to three-month follow-up will be compared between the two randomised groups.

Insomnia symptoms will be measured by the Insomnia Severity Index (ISI) [27], which assesses insomnia symptom severity, distress and daytime impairment over the past two weeks. Each of the seven items are assessed on a likert-based scale which provide an overall quantitative score ranging from 0 to 28, with higher scores indicative of more severe insomnia. The tool has been shown to be a valid and reliable measure [27] and is sensitive to change following intervention for primary insomnia in older adults [40]. In the current investigation, mean changes in continuous insomnia symptom scores from baseline will be compared between the two randomised groups at three-month follow-up.

#### Tertiary outcomes

Changes in actigraphic and sleep diary assessed sleep parameters (total sleep time, sleep efficiency, wake after sleep onset, sleep onset latency), hypnotic use, sleep beliefs (Dysfunctional Beliefs about Sleep (DBAS); [41]), the clinician rated scales above and self reported health related quality of life (Medical Outcomes Study Short-Form Health Survey; SF-12) will be measured at baseline and post-intervention. In addition, user characteristics predictive of outcome such as demographic, health and lifestyle factors will be described by treatment group at trial entry and overall at three and six months. Program adherence and drop-out will be monitored throughout the intervention period, and evaluation measures will be measured at post-intervention.

### Participant timeline

Potential participants will visit the trial-specific website www.somna.com.au where they will be provided with detailed information about the trial, the procedures and time commitment involved in participation, the randomisation process, and confidentiality, and contact details for the trial team should they want further information. The Participant Information Statement and Consent Form will be published on the website and available for download. Those interested in participating in the trial will undertake an online consent and preliminary eligibility screening procedure.

Completion of online consent and preliminary eligibility screening requires potential participants to provide their full name and contact details, along with their date of birth, gender and confirmation of their regular access to an internet-enabled computer or device and availability to attend the Trial Coordinating Centre on at least four occasions. Potential participants will then be asked general information pertaining to current sleep habits, and to complete the QIDS-SR, ISI and RLS screening tools. Interested participants are asked to sign the consent form by dragging their mouse to create their signature, thus giving permission to the research team to assess their preliminary screening responses and contact them to conduct an interview over the telephone to further confirm eligibility to participate. This process also creates a unique profile (trial identification number) for the potential participant within the trial website.

Upon submission of the online consent and preliminary screening questionnaire, a member of the SOMNA trial team will assess the potential participant’s eligibility against the trial inclusion criteria. Those that do not meet criteria will receive an email outlining why they have not been selected to participate and will be provided with a resource sheet outlining options for alternative treatment or support for depression and sleep concerns. A research assistant will contact those who meet trial inclusion criteria via telephone, and an interview assessment against the trial exclusion criteria will be completed. Participants who do not meet criteria, due to prior diagnosis of psychosis or hypo/manic episodes, high risk or prior diagnosis of sleep apnea, or who report they are a rotating shift worker, will be thanked for their time and will not participate further. If required further referral information will be provided. If potential participants report recent transmeridian travel, they will be invited to undergo all preliminary depression and insomnia screening questions again once they have had opportunity to readjust to the present time zone. Wherever psychiatric history is unclear, participants will be invited to attend for baseline assessment whereby the trial psychiatrist will undertake a comprehensive clinical interview to confirm diagnosis.

Following telephone screening, all eligible participants will be invited to attend the Trial Coordinating Centre (Brain & Mind Research Institute, Sydney, Australia) to commence baseline assessment. Whilst at the Trial Coordinating Centre, the participant will complete the baseline medical assessment and clinical interview and an online self-report questionnaire. Ongoing participation in the trial will be measured from this time-point. As part of the baseline assessment, participants will be provided with an actigraphy watch to wear on their wrist for the subsequent two weeks, and they will be instructed to log into the SOMNA website each day during this period to submit sleep diaries. Upon completion of ten diaries in a 14-night period the participant will be eligible to commence the active/control intervention. Full informed, written, consent will be obtained prior to any study procedure being undertaken.

Upon completion of the nine-week intervention period, participants will be prompted by automated email to return to the trial website to complete the post-intervention self-report questionnaire. Personalised emails, text message reminders and telephone calls will be made to participants who do not respond to encourage completion. Participants will also be invited to return to the Trial Coordinating Centre to complete the post-intervention medical assessment, and have the option of completing the online questionnaire at that time too. As per the baseline assessment, they will be provided with an actigraphy watch to wear on their wrist for the subsequent two weeks, and they will be instructed to log into the SOMNA website each day during this period to submit sleep diaries.

A final follow-up assessment will be completed six-months later, although on this occasion only self-report measures will be collected through the study website. Personalised emails, text message reminders and telephone calls will be made to participants who do not initially respond. Refer to Figure 1 for further detail regarding the timeframe for follow-up assessments.

Where participants are unable to complete follow-up self-report assessments online, a hardcopy of the assessment will be mailed to them for completion at home. Alternatively, a blinded research assistant may complete the assessment over the telephone. Where a participant discontinues their assigned intervention, all efforts will be made to retain the participant in the trial to enable follow-up data collection and prevent missing data.

### Sample size

In older adults, literature suggests depression treatment comprising pharmacotherapy or psychotherapy will elicit medium effects on symptomology. In the secondary analysis of the primary SHUTi trial, the effect of the intervention on depressive symptoms was larger than this, with a reported effect size on reducing depressive symptoms of 0.72 pre and post assessment using the Beck Depression Inventory [22]. Although noting this was detected amongst a community sample with mild depressive symptoms, we hypothesise that an adjunctive SHUTi intervention will lead to a small to moderate effect size over and above the effect of standard guideline-based depression treatment in a naturalistic setting. We propose to conduct a randomised controlled trial that will seek to establish the true effect size, along with feasibility of conducting a definitive clinical trial in a large-scale primary care setting that would provide evidence on the potential benefit of an adjunctive insomnia intervention for depression amongst older men. We will attempt to recruit a minimum of 80 participants with 40 per group (36 per group with 10% attrition) for the comparison of endpoint depression scores between the SHUTi intervention and the control condition.

### Recruitment

Trial participants will be recruited into the study on a rolling basis over a period of 18-months. The entire study is expected to be complete within three years.

### Allocation

The allocation of participants to study arm will be undertaken by a staff member who is independent of the study operations and has no day-to-day contact with participants. Once full informed consent is obtained and the trial psychiatrist has assessed eligibility and made the decision to enrol a participant, a research assistant will notify the independent staff member to perform the random allocation. Treatment allocation will be undertaken in accordance with the electronic randomisation schedule, which will be generated by the unblinded trial manager prior to trial commencement, password-protected and saved on a secure network. In addition to documenting each allocation on the randomisation schedule, so as to generate a unique username and password to enable each randomised participant access to their allocated website program, the independent staff member will be required to assign the allocated program to the user’s profile in the trial website.

### Sequence generation

The allocation sequence will be generated using a randomly generated number sequence calculated used Research Randomizer (http://www.randomizer.org/) prior to trial commencement. The fully replicable sequence will be based on permuted blocks, and eligible participants will be stratified according to presenting depression severity (their summed HAM-D score obtained during the baseline medical assessment and clinical review) to ensure participants with higher levels of symptoms are distributed equally across both conditions. In accordance with severity classification that maximises the sensitivity and specificity of the HAM-D scale, symptom severity will be collapsed into two categories including *mild-moderate* scores <17; *moderate-severe* scores ≥17 [42]. The allocation sequences within each strata will be pre-generated by the unblinded trial manager and documented in the randomisation schedule prior to trial commencement.

### Allocation concealment mechanism

The allocation codes will be saved in a password-protected file accessible only to the independent staff member who performs randomisation and the unblinded trial manager, not to be provided until the completion of the trial, unless unblinding is required in an emergency. The allocation code assigned to any participant will only be broken by the Chief Investigator or trial manager if the patient’s safety is at risk and it is absolutely necessary to ascertain the website program they were allocated. Given the low risk of the intervention under study, this scenario is unlikely to eventuate. However, where patient safety is under consideration, disabling access to the website program will be the preferred management option in contrast to unblinding.

### Blinding (masking)

This is a double-blind clinical trial whereby the study participants and the clinical investigators / trial monitoring staff, including the treating psychiatrist, will be unaware of treatment assignment. Only the trial manager, the Internet program system administrator and the trial statistician will have access to unblinded data at the individual level. However, they will not have any direct access to trial participants.

Several strategies will be employed to minimise bias through accidental unblinding. Knowledge of treatment allocation will be minimised by virtue of the intervention delivery method being web-based, along with all self-report assessments. Personnel who are unaware of the participant’s treatment allocation will assess all clinical outcomes. On each occasion of clinical assessment, participants will be reminded that staff must remain blinded to treatment allocation, and as such they should refrain from describing or discussing their website program. In the event that treatment allocation is accidentally revealed to a staff member during an assessment, an alternative assessor, who is blind to allocation, will conduct subsequent follow-up assessments. The trial manager will maintain an unblinding log to record any specific personnel that may be accidentally (or deliberately) unblinded during the course of the trial, along with the date, scope and reason for doing so.

### Data collection methods

Reasons for exclusion, withdrawal, discontinuation of the website program or the depression treatment plan, or loss-to-follow-up will be recorded. Table 1 provides an overview of the timeframe for all assessments and the measures that are used. All self-report measures, including sleep diary entries, will be collected via the study website at baseline, three and six-month time-points.

**Table 1:** **Complete list of assessments and measures**

	**Recruitment/screening**	**Baseline assessment**	**Visit 1**	**Monitoring calls**	**Post-intervention follow-up assessment**	**Post-intervention follow-up assessment**	**6 Month post-intervention follow-up assessment**	**Early termination**
**Time-point (Week)**	**- 4**	**1**	**3**	**4 and 8**	**12**	**14**	**36**	
**Assessments**								
Informed consent	**X**	**X**						
Inclusion/exclusion criteria	**X**	**X**						
Self report measures		**X**		**X**	**X**		**X**	
Sleep diary		**X**			**X**		**X**	
Actigraphy		**X**			**X**			
Psychiatric assessment		**X**			**X**			**X**
Medical check-up*		**X**	**X**		**X**			
Monitor for suicidality*		**X**	**X**		**X**			**X**
Adverse events*			**X**		**X**			**X**
**Forms/measures**								
Depression (QIDS)	**X**							
Restless legs (RLS)	**X**							
Insomnia (ISI)	**X**	**X**		**X**	**X**		**X**	
Obstructive sleep apnea (Berlin questionnaire)	**X**							
Psychiatric diagnosis (SCID)		**X**			**X**			
Global functioning (GAF)		**X**			**X**			
Depression (HAM-D)		**X**	**X**		**X**			
Anxiety (HAM-A)		**X**			**X**			
Disease burden (CIRS-G)		**X**						
Social and occupational functioning (SOFAS)		**X**			**X**			
Cognition (MMSE)		**X**						
Depression (CES-D)		**X**		**X**	**X**		**X**	
Anxiety (STPI)		**X**			**X**		**X**	
Sleep (DBAS)		**X**			**X**		**X**	
Sleep (MEQ)		**X**						
Health and wellbeing (SF-12)		**X**			**X**		**X**	
Alcohol use (AUDIT)		**X**			**X**		**X**	
Physical activity		**X**			**X**		**X**	
Personality (Locus of control)		**X**						
Program evaluation					**X**			

*Additional clinical reviews will be scheduled on an individual basis in accordance with personalised depression treatment plan.

Key to Abbreviated Forms/Measures: QIDS (Quick Inventory of Depressive Symptomatology); RLS (Restless Legs Syndrome Rating Scale); ISI (Insomnia Severity Index); SCID (Structured Clinical Interview for DSM-IV Disorders); GAF (Global Assessment of Functioning); HAM-D (Hamilton Rating Scale for Depression); HAM-A (Hamilton Anxiety Rating Scale); CIRS-G (Cumulative Illness Rating Scale for Geriatrics); MMSE (Mini Mental State Examination); CES-D (Center for Epidemiologic Studies Depression Scale); STPI (State Trait Personality Inventory); DBAS (Dysfunctional Beliefs About Sleep Scale); MEQ (Morning-Eveningness Questionnaire); SF-12 (Short Form 12-Item Survey); AUDIT (Alcohol Use Disorders Identification Test).

All clinical (medical, psychiatric and actigraphy) data will be collected using standardised instruments and tools by staff trained in the trial protocol and procedures at baseline and three-months. All medical and psychiatry assessments will be performed at the Trial Coordinating Centre, whereas actigraphy data will be collected via the watch worn by participants in their homes. Clinicians will be given a hardcopy case record form (CRF) in which they will document results, including actigraphy, which will be downloaded when the watch is returned. Clinicians will be required to sign-off on the CRF on completion of each assessment.

### Data management

All consenting participants will be allocated a unique identifier at the time of preliminary screening and this identifier will be used exclusively to link all data collected as part of the trial. All self-report data will be collected via the secure internet site that delivers both arms of the treatment program (intervention and control). This data will be held on a secure server maintained by the provider of the internet-delivered interventions, BeHealth Solutions, and will only be transferred to the secure master trial database at the Trial Coordinating Centre at trial-end.

All clinical data (medical, psychiatric and actigraphy) will be entered electronically into the master trial database by suitably qualified staff, supervised by the trial manager. Data will be entered into a user-friendly Filemaker platform and integrity will be enforced through a variety of mechanisms, including referential data rules, valid values, range checks and consistency checks. The option to choose a value from a list of valid codes will also be available when applicable. The master trial database will be held on a secure server hosted by the Faculty of Medicine, University of Sydney. The trial database will be password protected, and only authorised trial personnel will have access. The database will be maintained in accordance with University of Sydney Information and Communication Technology policy and procedures. Upon database lock, data will be exported to a statistical software package for subsequent analysis.

### Statistical methods

Primary outcome analysis will be undertaken on an intention-to-treat basis. Mixed-model repeated measures analyses will be used because of the ability of this approach to include participants with missing data. Characteristics of the participants including sociodemographics, physical activity, health and lifestyle factors will also be described by treatment group and overall. Safety criteria including adverse events and withdrawals will also be analysed. The type one error will be set at 5%. Descriptive statistics will include number of participants (N), mean, standard deviation, minimum, maximum and if a non- normal distribution, the median and first/third quartiles will be reported.

### Data monitoring

The accuracy, completeness and progress of data will be monitored continuously throughout the trial. Each CRF will have a data checklist attached, on which the assessments completed and other information will be recorded. Any missing information or illegible data will be queried with the researcher, clinician or interviewer responsible for its collection. Audits will be undertaken periodically to ensure that all data relating to informed consent, self-report questionnaires, medical and psychiatric assessment, and actigraphy, are accounted for, and that psychiatric assessments are standardised. All original CRFs will be stored securely by trial identification number at the Trial Coordinating Centre.

Following data entry into the master trial database, data will be randomly checked for accuracy and completeness and verified against source data. Approximately 5% of participants will be specifically monitored.

### Safety and event monitoring

The intervention under study is considered low-risk, with the only safety concern associated with the active SHUTi intervention related to increased tiredness and its sequelae due to restricted time spent in bed (as per the behavioural treatment for insomnia). This risk is mitigated directly through the program, which does not restrict sleep by fewer than five hours. In light of this, and given that all trial participants will be under the clinical care of their treating psychiatrist throughout the intervention period, an independent Data and Safety Monitoring Committee will not be established.

### Harms

During the course of the study, participants will be assessed for both expected and unexpected adverse events (AEs) at each clinical and research assessment time point. Adverse events will be defined as any unfavourable and unintended sign, symptom or disease temporarily associated with the use of the intervention under investigation. Medical conditions or diseases present before starting the intervention will be considered adverse events only if they worsen after starting the trial and that worsening is considered to be related to the study intervention. An AE will also be extended to include any undesirable and unintended effect of research occurring in trial participants as a result of the collection of identifiable private information under the research. An AE will be deemed serious if it is determined to be associated, or possibly associated with study involvement, and results in either: death, is life-threatening, requires or prolongs hospitalisation, or results in persistent or significant disability or incapacity. Expected AEs will include: initial discomfort or embarrassment due to personal or sensitive nature of research questions; concerns related to internet-based data collection and storage procedures; and initial tiredness, fatigue and possible concentration problems as a result of sleep restriction techniques to improve sleep habits.

Adverse events will be detected at the Week 3 clinical review and Week 12 follow-up assessment. In addition, research assistants conducting routine monitoring telephone interviews at Weeks 4 and 8, will also have capacity to detect AEs.

All AEs (including Serious Adverse Events, or SAEs), regardless of relatedness to the interventions under investigation, will be recorded, classified and graded on an adverse event form. Recording will be completed in a concise manner using standard, acceptable medical terms. All SAEs will be reported to the Human Research Ethics Committee within 72 hours.

### Study sponsorship and organisation

The sponsor of the trial is the University of Sydney. SHUTi and the control program will be provided by BeHealth Solutions. The trial is supported by *beyondblue: the national depression and anxiety initiative* National Priority Driven Research Program and funded through a donation from the Movember Foundation. The trial will be coordinated independently of the sponsor, funder and supplier of the intervention programs, by the Brain & Mind Research Institute, Sydney, Australia and overseen by the Trial Management Committee comprising the Chief Investigator team.

### Ethical considerations

The trial will be undertaken in compliance with the World Medical Association Declaration of Helsinki (revised version of Seoul, 2008), international standards of Good Clinical Practice and the applicable regulatory requirements in Australia. The design and implementation of the trial has been approved by The University of Sydney Human Research Ethics Committee (Reference Number: 2012/2182). All important protocol modifications will be communicated to the Human Research Ethics Committee prior to implementation.

## Discussion

This RCT will provide evidence on whether a widely accessible, evidence-based, internet-delivered CBTi intervention can also improve other clinical outcomes in a population receiving standard treatment for depression, which traditionally do not readily access current care systems. The outcomes may also be readily applicable for translation into health care in other settings. Finally, the study will provide the required information to inform the conduct of a definitive RCT.

## Abbreviations

ANZCTR: Australian and New Zealand Clinical Trials Registry
AE: Adverse Event
AUDIT: Alcohol Use Disorders Identification Test
CRF: Case Record Form
CBTi: Cognitive Behavioural Therapy for Insomnia
CES-D: Center for Epidemiologic Studies Depression Scale
CIRS-G: Cumulative Illness Rating Scale for Geriatrics
DBAS: Dysfunctional Beliefs About Sleep Scale
DSM-IV: Diagnostic and Statistical Manual of Mental Disorders
GAF: Global Assessment of Functioning
GP: General Practitioner
HAM-A: Hamilton Rating Scale for Anxiety
HAM-D: Hamilton Rating Scale for Depression
ISI: Insomnia Severity Index
MDD: Major Depressive Disorder
MEQ: Morning-Eveningness Questionnaire
MMSE: Mini Mental State Examination
OSA: Obstructive Sleep Apnea
QIDS-SR: Quick Inventory of Depression Symptomology Self Report
RCT: Randomised Controlled Trial
RLS: Restless Legs Syndrome
SAE: Serious Adverse Event
SCID: Structured Clinical Interview for DSM-IV
SF-12: Medical Outcomes Study Short-Form 12-Item Health Survey
SHUTi: Sleep Healthy Using the Internet
SOFAS: Social and Occupational Functioning Assessment Scale
SOMNA: Sleep Or Mood Novel Adjunctive therapy
STAR*D: Sequenced Treatment Alternatives to Relieve Depression Study
STPI: State Trait Personality Inventory
